# Advances in PGD2/PTGDR2 signaling pathway in tumors: A review

**DOI:** 10.17305/bb.2024.10485

**Published:** 2024-10-01

**Authors:** Hengjin Tian, Kunpeng Ge, Lulu Wang, Peiyao Gao, Amin Chen, Feifan Wang, Fangzheng Guo, FengChao Wang, Qiang Zhang

**Affiliations:** 1 Department of Clinical Laboratory, The First Affiliated Hospital of Bengbu Medical University, Bengbu, China; 2 Key Laboratory of Cancer Research and Clinical Laboratory Diagnosis, Bengbu Medical University, Bengbu, China; 3 Department of Blood Transfusion, The First Affiliated Hospital of Naval Medical University, Shanghai, China; 4 Anhui Province Key Laboratory of Immunology in Chronic Diseases, Bengbu Medical University, Bengbu, China

**Keywords:** Prostaglandins (PGs), prostaglandin D2 (PGD2), PGD2 receptor (PTGDR2), inflammation, cancer, signaling pathway

## Abstract

Studies have shown that the prostaglandin (PG) family acts as an allergic inflammatory mediator in malignant diseases. Furthermore, prostaglandin E2 (PGE2) and its related receptors, as well as the prostaglandin D2 (PGD2)/PGD2 receptor (PTGDR2), play irreplaceable roles in tumorigenesis and anti-tumor therapy. Several experiments have demonstrated that PGD2 signaling through PTGDR2 not only directly inhibits cancer cell survival, proliferation, and migration but also reduces resistance toward conventional chemotherapeutic agents. Recent studies from our and other laboratories have shown that PGD2, its ligands, and related metabolites can significantly alter the tumor microenvironment (TME) by promoting the secretion of chemokines and cytokines, thereby inhibiting tumor progression. Additionally, reduced PGD2 expression has been associated with poor prognosis in patients with gastric, breast, lung, and pancreatic cancers, validating the preclinical findings and their clinical relevance. This review focuses on the current understanding of PGD2/PTGDR2 expression patterns and biological activity in cancer, proposing questions to guide the assessment of PGD2 and its receptors as potential targets for effective cancer therapies.

## Introduction

**Figure 1.**
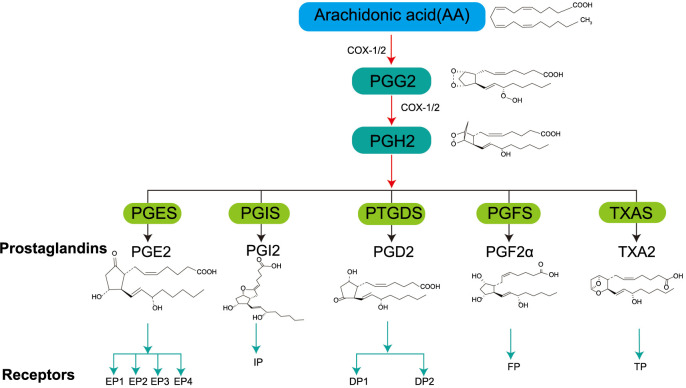
**PGD2 biosynthetic pathway.** PGH2: Prostaglandin H2; PTGDS: PGD2 synthase; PGE2: Prostaglandin E2; PGES: PGE2 synthase; PGF2α: Prostaglandin F2α; PGFS: PGF2α synthase; PGD2: Prostaglandin D2; PGG2: Prostaglandin G2; PGI2: Prostacyclin; PGIS: PGI2 synthase; TXA2: Thromboxane A2; TXAS: TXA2 synthase; COX: Cyclooxigenase; IP: Prostacyclin receptor; FP: Prostaglandin F receptor; TP: Thromboxane receptor.

**Figure 2.**
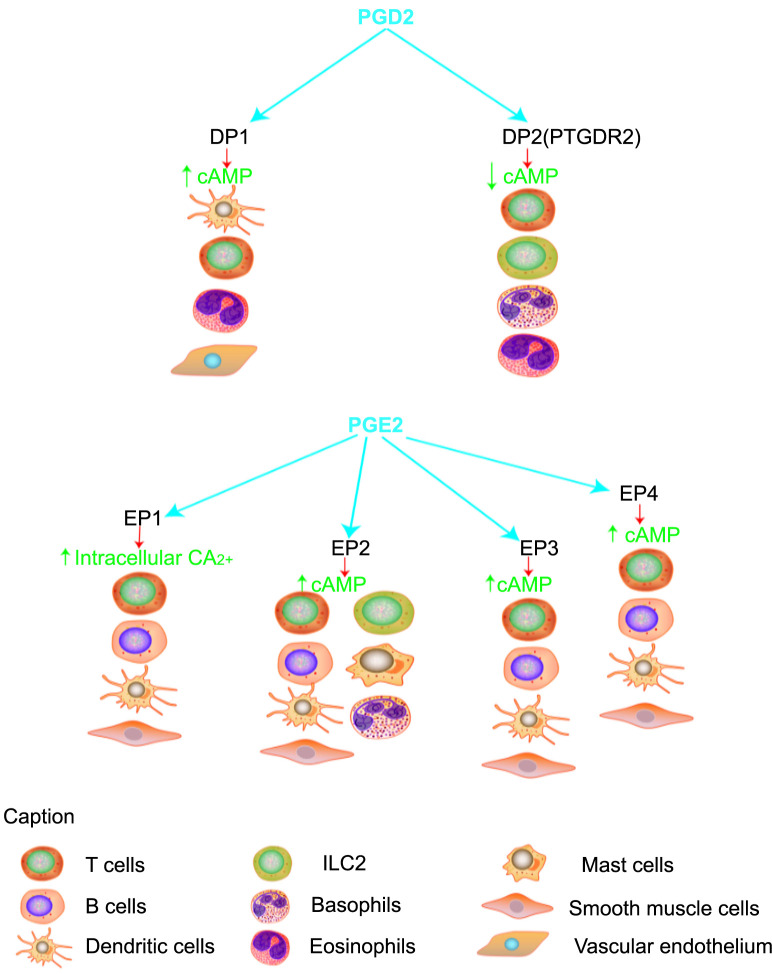
**PGD2 and PGE2 bind to ligands via signal transduction to increase or decrease cAMP.** PGD2: Prostaglandin D2; PGE2: Prostaglandin E2; cAMP: Cyclic adenosine monophosphate; ILC2: Type 2 innate lymphoid cells.

Prostaglandins (PGs) are biologically active endogenous metabolites derived from arachidonic acid [1], which is catalyzed by cyclooxygenase (COX) to produce an intermediate called prostaglandin H2 (PGH2). Subsequently, in the presence of other dehydrogenases, PGH2 is converted to other PGs, such as prostaglandin E2 (PGE2), prostacyclin (PGI2), prostaglandin D2 (PGD2), prostaglandin F2α (PGF2α), and thromboxane-A2 (TXA2), which then act through different G protein-coupled receptors (GPCRs) [2] (Figure 1). There are two isoforms of COX: COX-1 is the constitutive isoform that produces PGs, which maintain homeostasis and protect kidney and gastric tissues from damage [3]. Whereas COX-2 is the inducible isoform stimulated by inflammatory factors and produces PGs, which promote inflammatory response or form a tumor microenvironment (TME) [4, 5]. Increased levels of PGs have been detected in tracheal alveoli of acute respiratory distress syndrome patients [6]. Some studies have found that the use of COX-2 inhibitors in animal models prevented sepsis and increased its survival rate [7]. The above studies indicate that COX-2-mediated PG production is critical for the onset and progression of inflammation. The binding of PGs with their receptors activates the receptor and induces heterotrimeric G-protein complexes dissociation, thereby generating distinct signaling cascades [8]. PGE2 synthase catalyzes PGH2 to produce PGE2, a COX-2 product. PGE2 binds to its cognate G protein receptor family members (EP1, EP2, EP3, or EP4) to activate relevant signaling pathways that mediate inflammatory, neoplastic, and related immune responses [9] (Figure 2). For example, PGE2 binding with EP1 activates protein kinase C (PKC)/nuclear factor-κB (NF-κB)/human forkhead box protein C2 (FOXC2) and EGFR/PI3K signaling pathways, which increases intercellular adhesion molecule-1 (ICAM-1), thereby causing tumor growth and migration [10–12]. Whereas PGE2/EP2 binding promotes the expression of inflammatory factors, such as IL-1β and IL-6 [13]. Furthermore, PGE2 interacts with EP3/EP4 and activates ERK1/2, p53, and PKA/PI3K/AKT signaling pathways, which promote cancer cell proliferation, migration, and growth [9, 14]. Moreover, PGE2 not only increases tumor proliferation, migration, and associated angiogenesis via related pathways, but it also reprograms cells (e.g., myeloid cells) to become tumor-associated macrophages (TAMs), thus promoting the invasive growth of tumor cells [15, 16]. PGD2 synthase (PTGDS) catalyzes PGH2 and produces PGD2, which is also a COX-2 metabolite and PG member and mediates inflammatory response [17]. It was initially thought to be associated with allergic diseases in humans, and its aberrant expression was markedly linked with many human diseases, especially inflammation, and tumors. For instance, PGD2 stimulates human peripheral blood type 2 innate lymphoid cells (ILC2) and participates in inflammatory responses by producing IL-13 via the PGD2 receptor (PTGDR2) signaling pathway [18]. PTGDR2 receptors are involved in the activation and migration of helper T-cell type 2 (Th2), lymphocytes, eosinophils, and basophils, as well as the synthesis of cytokines, such as IL-4, IL-13, etc. [19]. The results from the above studies indicate that the PGD2/PTGDR2 signaling pathway plays an important role in inflammation, and according to recent research, this pathway can inhibit cancer cell growth, proliferation, and migration [20–22]. Low expression of PGD2 and PTGDR2 is associated with poor prognosis in different types of cancer. This review first discusses the expression pattern and biological functions of PGD2 inflammatory mediators and then focuses on their implication in tumorigenesis highlighting gaps that remain in our understanding of this topic.

## Prostaglandin D2 (PGD2) and its prostaglandin D2 receptor (PTGDR2)

### PGD2 expression

The PGD2 is synthesized by COX and PTGDS, mainly in mast cells and macrophages [23]. PTGDS has two isozymes: lipocalin-type PTGDS (L-PTGDS) and hematopoietic PTGDS (H-PTGDS). The L-PTGDS is predominantly expressed in the central nervous system and is involved in sleep regulation [24]. Whereas H-PTGDS primarily promotes PGD2 biosynthesis in mast cells and Th2 lymphocytes, which are involved in asthma and inflammatory responses, therefore, H-PTGDS is a potential target for the treatment of asthma and allergic inflammation [25, 26]. Both PGD2 and PGE2 are synthesized from PGH2, where PGE2 mediates inflammation and tumor development [4, 26]. Recent research has indicated that PGD2 inhibits vascular permeability in acute lung inflammation and tumor models, as well as alleviates contact hypersensitivity induced by 2,4,6-trinitrochlorobenzene and peritonitis induced by yeast glycan- in mice [23, 27, 28]. These data indicate that PGD2 has an anti-inflammatory effect primarily. PGD2 performs most of its function via GPCRs D prostanoid 1 (DP1), the chemoattractant receptor homolog molecule (CRTH2) expressed on Th2 cells (i.e., PTGDR2), and the thromboxane PG-like (TP) receptor [29]. DP1 receptors are primarily expressed on the surface of certain leukocytes, including eosinophils, dendritic cells (DCs), and other cells, and produce active molecules [27]. Furthermore, activation of the DP1 receptor stimulates adenylate cyclase and increases intracellular cyclic adenosine monophosphate (cAMP) levels [28] (Figure 2). Moreover, PGD2/DP1 receptor binding promotes vasodilation, bronchodilation, and anti-inflammatory effects by inhibiting the chemotaxis of eosinophils, basophils, and DCs [25, 30–32]. 

In tumors, the activation of PGD2/DP1 signaling enhances endothelial barrier function and inhibits tumor angiogenesis to exert anti-tumor effects [33]. PTGDR2 receptors are described in the next section. In humans, the TP receptor is a GPCR encoded by the thromboxane-A2 R gene and expressed predominantly on the surface of platelets, monocytes, macrophages, and endothelial cells (ECs). Furthermore, TP activation contracts bronchial smooth muscles [34–36]. The 15-deoxy-Δ 12,14 prostaglandin J 2 (15-d-PGJ-2) is a PGD2 metabolite and a natural ligand for peroxisome proliferator-activated receptor-γ (PPARγ) which is activated by dependent or non-dependent signaling and exert anti-tumor, anti-inflammatory, and anti-angiogenic actions [36, 37]. Additionally, PPARγ signaling activation regulates inflammatory responses and cell differentiation as well as promotes apoptosis via NF-κB signaling, transcription activator factor (STAT)-1, and activator protein-1 (AP-1) signaling [36]. Moreover, PGD2 inhibits tumor angiogenesis and promotes tumor cell necrosis by downregulating the expression of vascular endothelial growth factor (VEGF) as well as its receptors FLT-1 and FLK/KDR. Furthermore, PGD2 and 15-d-PGJ-2, also reduce PGE2 synthesis by inhibiting COX-2 and PGE2 synthase, thus exerting anti-inflammatory and anti-tumor effects [37, 38].

### PGD2 receptor expression

The PTGDR2 is a chemoattractant receptor homolog molecule (CRTH2) expressed on Th2 cells, also known as GPR44, DP2, PTGDR2, or CD294 [39, 40]. It was originally identified as a GPCR on human Th2 cells and is the most reliable Th2 cell surface marker [40]. The gene that encodes the PTGDR2 receptor is located on the long arm of chromosome 11 (11 q12), has a molecular weight of 43 kDa, comprising 395 amino acids, and is a member of the GPCR (seven-transmembrane receptor family) [19]. The binding affinity of PGD2 for PTGDR2 is about 8-fold less than that of the DP1 receptor, therefore, its rate of dissociation is much faster [41]. PTGDR2 has two N-glycosylation sites in the extracellular domain of the N-terminal and a characteristic long cytoplasmic tail region, which also contains multiple PKC phosphorylation sites, thought to be responsible for the desensitizing effect of the receptor [42, 43]. Previous literature has also indicated the presence of PTGDR2 in various body tissues at the mRNA level and recently it was also found to be expressed in eosinophils, basophils, monocytes, as well as in the T-cell subpopulations CD4+ Th2 and CD8+ Tc2 cells [42]. Interestingly, the protein and mRNA expression of PTGDR2 are rare in neutrophils [43, 44]. Moreover, PTGDR2 is currently the only lipid receptor linking mast cells to Th2 cells, eosinophils, and basophils [43]. Overall, these data indicated that PTGDR2 is closely associated with various immune cells, suggesting its crucial role in immunity [18]. PTGDR2 activation requires the recruitment of Th2 cells and other leukocytes by PGD2, which subsequently induces Th2 cytokines IL-4, interleukin-5 (IL-5), and IL-13 [45]. In addition, PTGDR2 inhibits adenylate cyclase via Giα protein, thereby reducing intracellular cAMP levels, activating immune cells, and exerting biological effects [25, 46].

### PTGDR2 signaling pathway

Currently, research on the PTGDR2 signaling pathway is limited, and only a few studies have reported that PTGDR2 competitively binds STAT3 with IL-6 R or Janus kinase 2 (JAK 2), thereby affecting STAT3 phosphorylation levels [20], which reduces IFN-γ (IL-28 A) production upon ligand binding [47]. Furthermore, PTGDR2 exerts anti-inflammatory and anti-tumor effects by inhibiting the classical NF-κB signaling pathway as well as KRAS, MAPK, ERK1/2, and Akt-mediated transcription factor signaling pathways [48–51].

### PGD2/PTGDR2 pathway in allergic and inflammatory diseases

PGD2 released by desensitized mast cells during exogenous allergen stimulation is thought to be a key molecule involved in asthma and allergic responses [19]. Furthermore, it is also associated with microbial infections, cardiovascular, cancer, and other diseases [33–36]. Moreover, PGD2 has been observed to be essentially involved with allergic reactions, evident from the increased inflammation and cytokine release from eosinophils in the lungs, such as leukotriene C4 (LTC 4) [52, 53]. In addition, PGD2 induces eosinophil chemotaxis via its receptor PTGDR2, which upregulates cell migration and adhesion molecules and participates in the body’s allergic response [54]. Additionally, PGD2 has been indicated to enhance immune-mediated basophils and histamine release, which exacerbates allergic reactions. PTGDR2 exogenous agonists administration in animal models induces eosinophilic infiltration into lung and skin tissues, which also exacerbates the allergic response [55, 56]. It has been observed that in a mouse model of endotoxin-induced acute lung injury (ALI), PTGDR2 activation causes early polarisation of alveolar macrophages, causing neutrophil recruitment and increased lung inflammation. Whereas its antagonists ameliorate allergen-induced skin, lung, and respiratory inflammation [57–60]. Increased PGD2 levels have been detected in the bronchoalveolar lavage fluid (BALF) of severe asthma patients [61, 62]. In summary, these results illustrate the pro-inflammatory role of PGD2 and PTGDR2. 

Diwakar et al. [48] found that PTGDR2 knockdown enhanced the inflammatory response in macrophages and peritoneal cells in a mouse model, while mice with bleomycin-induced pulmonary fibrosis showed higher mortality, increased inflammatory response, and collagen deposition [49]. This indicates the anti-inflammatory efficiency of PTGDR2, and that certain lipid mediators, such as lipoxins, can inhibit the infiltration of inflammatory cells [63]. Furthermore, Murata et al. [7] found that PGD2 can stimulate lipoxin production and, thus, exert an anti-inflammatory effect. In inflammatory bowel disease, increased PGD2 levels can reduce inflammatory cell infiltration in the tissue mucosa, providing a protective effect in patients [64]. Consistently, Syed et al. [65] studied a mouse model of sepsis and found that PGD2 modulated the expression of triggering receptor (a hyperimmunoglobulin receptor found on macrophages and neutrophils, TREM-1) on myeloid cells-1 in macrophages by activating nuclear factor-E2-related factor 2 (Nrf-2) and inhibiting transcription factors, such as NF-κB, as well as improve survival and reduce inflammation in mice. It has been observed that PGD2 overexpression ameliorates *Pseudomonas aeruginosa* infection and protects mice [66]. In another anti-inflammatory pathway, PGD2 can directly inhibit vascular permeability and reduce tissue damage by inflammatory cells through DP1 receptor binding. Furthermore, 15-d-PGJ-2 mediates cytokine expression, inhibits neutrophil migration, and exerts antioxidant effects via the Nrf-2-mediated pathway. Moreover, 15-d-PGJ-2 has also been indicated to reduce inflammatory symptoms and protect against tissue damage in ALI induced by carrageenan (an inflammation inducer) in a rat pleurisy model [67, 68]. Genovese et al. [69] showed that administration of the exogenous PPARγ agonist, rosiglitazone or 15-d-PGJ-2, attenuated bleomycin-induced lung injury in mouse models. In addition, Rajakariar et al. [68] revealed that in vivo 15-d-PGJ-2 attenuated yeast polysaccharide-induced peritonitis in mouse models. Additionally, 15-d-PGJ-2 could protect rat lung tissues from inhalation injury and reduce infection- or allergy-induced lung inflammation by inhibiting the production of proinflammatory cytokines (TNF-α and IL-10) and gene expression of related chemokines (CCL2, CCL3, CCL4, and CXCL10) [70, 71]. 15-d-PGJ-2 also inhibits inflammation in gouty arthritis by decreasing oxidative stress levels and the expression of IL-1β, TNF-α, IL-6, IL-17, and IL-33 [72]. In addition to these diseases, upon nerve cell damage in the nervous system, PGD2 promotes the production of neurotrophic factors by astrocytes, thus providing neuroprotection in diseases, such as Parkinson’s and Alzheimer’s [73]. Overall, these results indicate that PGD2 plays a protective role in inflammation. Since inflammation can promote cancer progression, inhibiting inflammation can reduce tumor progression. PGD2 inhibits inflammation, therefore, it has an anti-tumor angiogenesis effect. Similarly, in mouse tumor models and patients, PGD2/PTGDR2 exhibits anti-tumor effects and PGE2 pro-tumor activity.

### Role of the PGD2/PTGDR2 signaling pathway in tumor expression and prognosis

Over the past decade, a lot of research has indicated the potential role of the PGD2/PTGDR2 signaling pathway in tumors, and that dysregulation of PGD2 expression and its receptor is associated with the prognosis of different types of tumors (Table 1). Zhang et al. [20, 21] studied 60 specimens of gastric cancer patients and revealed that the mRNA and protein levels of L-PTGDS and PTGDR2 were lower in gastric cancer tissues than in the normal tissues. Moreover, the down-regulated expression of PGD2 and PTGDR2 has been closely associated with poor prognosis and high mortality in gastric cancer patients. Bie et al. [74] indicated that yes-associated protein (YAP) could inhibit L-PTGDS and PTGDR2, which promotes stemness in gastric cancer stem cells (GCSCs), whereas PTGDR2 or L-PTGDS overexpression can block the YAP-induced self-renewal of GCSC. Fukuoka et al. [75] indicated that in 277 gastric cancer patients, 55% had PTGDR2 (including DP1) expression in cancerous tissues, which significantly correlated with lymph node metastasis, lymphovascular infiltration, and tumor, node, metastasis (TNM) stage. Furthermore, Zhang et al. performed ELISA on 178 plasma specimens (including 76 specimens from gastric cancer patients) and indicated significantly reduced plasma PGD2 levels in gastric cancer patients compared with patients with benign gastric lesions and healthy groups. Moreover, the PGD2 level was higher in poorly differentiated gastric cancer patients than in those with moderately and highly differentiated gastric cancer. These data indicated that plasma PGD2 levels correlated with the degree of differentiation of gastric cancer cells [21]. In addition, the microarray data analysis of 875 gastric cancer patients indicated that upregulation of PTGDR2 expression was significantly associated with a better prognosis. Lu et al. screened five key genes, including *PTGDR2, CXCL3, NPBWR1, TAS2R38,* and *ADCY6*, and their correlation with gastrointestinal tumor grades, related histological types, and patient age was assessed via the Gene Expression Omnibus (GEO) and The Cancer Genome Atlas (TCGA) database [76]. 

Wang et al. studied a cohort of 123 triple-negative breast cancer (TNBC) in addition to gastric cancer and constructed a column plot model using PTGDR2, which predicted the 1–5 year overall survival (OS) rate of these patients. It was found that the lower the expression of PTGDR2, the higher the malignancy and the worse the prognosis of breast cancer patients [77]. Similarly, Pan et al. [22] revealed that PGD2 and PTGDS expressions were negatively correlated with breast cancer, and the lower their expression was, the more invasive and proliferative the breast cancer was and the worse the patient’s prognosis. Subsequent microarray data analysis of 1089 breast cancer patients revealed that reduced PGD2 and PTGDR2 expression was linked with a worse prognosis and shorter OS. 

In lung cancer, the lack of PGD2 synthesized by H-PTGDS promotes the development of Lewis lung cancer, accelerates angiogenesis, and promotes inflammatory cell infiltration [29]. Another microarray data analysis of 504 lung cancer patients indicated that downregulated PGD2 and PTGDR2 expression was associated with a poorer prognosis and lower survival rate. McLemore et al. [78] analyzed 42 lung cancer patient specimens and revealed that PGD2 expression was downregulated in lung cancer tissues compared to normal lung tissues and that there was no significant difference between smoking and non-smoking lung cancer patients. 

Bioinformatics was used to analyze the expression of H-PTGDS and L-PTGDS, two synthetic enzymes of PGD2, which are less expressed in glioblastoma compared to normal brain tissue [79]. It was found that PGD2 inhibited the proliferation of human neuroblastoma cells and was more toxic to tumor cells than normal human cells [80]. It was observed by Payne et al. that PGD2 mRNA and protein levels were reduced in glioblastoma and significantly correlated with low patient survival. Furthermore, even after adjusting for tumor grade and patient age, the correlation remained statistically significant and could identify patients with a poor prognosis [81]. 

Recently, Lv et al. screened PTGDR2 as one of the key genes (of 853 genes), which was statistically significant for predicting OS or progression-free survival (PFS) in colon cancer patients [82]. Moreover, the bioinformatics analysis performed by Su et al. [83] revealed six key genes associated with colon cancer prognosis, including *SULT1B1, UGT2B15, PTGDR2, GPR15, BMP5*, and *CPT2*, and the higher expression of the genes in cancerous tissues, the longer the survival of colon cancer patients. In addition, Gustafsson et al. [84] examined PTGDR expression in 62 colon cancer patients and found that its expression was five times lower in cancerous tissues than in normal tissues. Microarray analysis of 1336 colorectal cancer patients showed that upregulated PTGDR2 expression was associated with a better prognosis and a higher patient survival rate. Yoshida et al. [85] found that PGD2 expression levels were significantly lower in the group with colon cancer liver metastasis compared to the group without liver metastasis, suggesting that detecting PGD2 levels can predict metastasis of the liver in colon cancer tissues. 

In addition, another microarray analysis of 177 pancreatic cancer patients revealed that patients with upregulated expression of PTGDR2 had a better prognosis and significantly better survival. Moreover, mouse models have indicated that cluster cell-derived PGD2 was expressed at lower levels in pancreatic tumor mice than in wild-type mice [86]. 

Additionally, PGD2 has been shown to inhibit prostate cancer progression. It has been indicated that PGD2 injection into prostate cancer mice model induces cancer cell apoptosis and improves the survival rate of mice [37]. Gao et al. [87] analyzed the expression of 28 relevant genes in 122 hepatocellular carcinoma (HCC) samples using qRT-PCR and revealed PTGDR2 as a significant independent predictor of recurrence. Subsequent bioinformatics analyses revealed that in 364 HCC patients, lower PTGDR2 expression was linked with shorter survival. 

Furthermore, in ovarian cancer, lower PGD2 protein and mRNA expression were significantly correlated with a higher risk of progression after chemotherapy. In a cohort of 114 patients with high-grade serous ovarian carcinoma (HGSOC), immunohistochemistry (IHC) analysis demonstrated that PGD 2 levels were strongly associated with disease-free survival (DFS), no recurrence, and sensitivity to platinum-based drug therapy. Additionally, regression analysis indicated PGD2 as an independent marker of good prognosis and recurrence [1]. Moreover, microarray analysis of 1435 patients revealed that downregulated PGDR2 was related to a worse prognosis.

In sarcoma, Munisamy et al. [88] detected the mRNA level of PTGDR2 and found that its dysregulation was associated with lower survival. Furthermore, oropharyngeal cancer patients with lower PGD2 expression had shorter OS. Additionally, it has been observed that the downregulated expression of PTGDR2, PTGDR1, and PTGIR genes was associated with a poorer prognosis in oropharyngeal cancer patients, and all three can be used to screen HPV-ctDNA-negative HPV oropharyngeal cancer patients [89]. 

The literature suggested that in addition to solid tumors, PTGDR2 (referred to as GPR44 in that article) activation in acute myeloid leukemia (AML) induces apoptosis of leukemic cells in a mouse model of human leukemia, a human AML cell line, and patient samples [51]. Furthermore, PTGDR2 knockdown causes splenomegaly and the appearance of more and larger leukemia cells in mice. Moreover, microarray analysis of 1608 AML patients revealed that upregulation of PTGDR2 expression was positively correlated with better patient prognosis and survival. 

Therefore, combined protein and bioinformatics analyses of gene expression datasets indicated an association between dysregulated expression of PGD2 or PTGDR2 and the prognosis of different cancer patients. However, PGD2/PTGDR2 signaling pathway’s anti-tumor and treatment resistance effects are mediated by different mechanisms that are not yet fully understood, as described in the following paragraph.

**Table 1:** Prostaglandin D2/prostaglandin D2 receptor expression in cancers

**Tumor (type)**	**Expression—prognosis**	**Mechanism—models**	**References**
Gastric	High expression of PGD2 and PTGDR2 is associated with a better prognosis	Upregulation of PTGDR 2 expression prevents STAT 3 phosphorylation and can inhibit the stemness of SGC-7901 and HGC-27	[20]
Gastric	High PGD2 expression is associated with a better prognosis	PGD2 and metabolites activate PPARγ signaling to inhibit gastric cancer cell growth, migration, and invasiveness	[116]
Gastric	The expression level of PGD2 positivley correlates with the prognosis of gastric cancer patients	PGD2/PTGDR2 signaling inhibits gastric cancer migration, and invasive ability and promotes apoptosis	[21]
Breast	The expression level of PGD2 is positively correlated with the prognosis of breast cancer patients, the higher the expression, the better the prognosis	PGD2 induces a decrease in TWIST 2 expression and interferes with angiogenesis in breast cancer by inhibiting ALDH1A1 expression	[22]
Breast	The expression level of PGD2 is positively associated with survival in breast cancer patients	Positive correlation between miR-155 levels and PGE 2/PGD2 ratio. miR-155 deletion induces upregulation of PGD2, which inhibits cancer cell proliferation and migration	[117]
Breast	Overall survival in triple-negative breast cancer can be predicted using five enriched cytokines, including PTGDR2		[78]
Glioblastoma	Reduced PGD2 expression is significantly associated with poor patient prognosis and survival	PGD2 deficiency non-induced enhanced proliferative activity of A172 glioblastoma cells	[82]
Colorectal	The expression level of PTGDR2 positively correlates with patient prognosis and survival, and the lower the expression of PTGDR2 in TNM stages I and II, the worse the prognosis of patients	PGD2 and 15 d-PGJ 2 have anti-tumor effects by activating PPARγ or inhibiting NF-κB signaling	[118]
Pancreatic	In pancreatic cancer tissues, PGD2 level expression positively correlates with patient prognosis	PGD2 reduces the expression of metalloproteinase MMP-2 and MMP-9 proteins thereby reducing their invasiveness and promoting apoptosis	[97]
Sarcoma	Significantly aberrant expression of four genes from the enrichment (including PTGDR2) was associated with lower overall survival in patients		[88]
Oropharyngeal	Enriched PTGDR1, PTGDR2, and PTGIR genes can be used to screen and evaluate oropharyngeal cancer		[89]
AML	Activation of PTGDR2 is associated with a better prognosis in leukemia patients	Activation of PTGDR2 in CyPGs to inhibit the Kras-mediated MAPK-PI3K/AKT/mTOR signaling pathway to promote apoptosis	[51]
Prostate	Upregulation of PGD2 expression in prostate cancer tissues is associated with higher patient survival compared to normal prostate tissues	PGD2 and 15 d-PGJ 2 activate PPARγ to exert anti-tumor effects and are involved in tumor microenvironment regulation	[119]
Liver	Enriched PTGDR2 can be a predictor of prognosis in hepatocellular carcinoma		[87]
Lung	The higher the expression of PGD2 in lung cancer tissues, the better the prognosis and the longer the overall survival of patients	PGD2 reduces auto TNF-α synthesis as well as anti-tumor angiogenesis	[29]
Ovarian	Upregulation of PGD2 expression positively correlates with better prognosis in ovarian cancer	Inhibition of the NF-κB signaling pathway by PGD2 reduces drug resistance and inhibits tumor metastasis	[1]
Melanoma	Higher PGD2 expression in malignant melanoma is associated with better prognosis	PGD2 inhibits aggressive tumor growth and angiogenesis	[105]

AML: Acute myeloid leukemia; PGD2: Prostaglandin D2; PTGDR2: Prostaglandin D2 receptor 2; PPARγ: Peroxisome proliferator-activated receptor γ; TWIST2: A tie-specific basic helix-loop-helix transcription factor; ALDH1A1: Aldehyde dehydrogenase 1A1; TNM: Tumor-node-metastasis; 15d-PGJ-2: 15-Deoxy-Delta-12,14-prostaglandin J2; NF-κB: Nuclear factor-κB; MMP-2/9: Matrix metalloproteinase-2 and 9; CyPGs: Cyclopentenone prostaglandins; TNF-α: Tumor necrosis factor-α.

### Mechanisms of action

**Figure 3.**
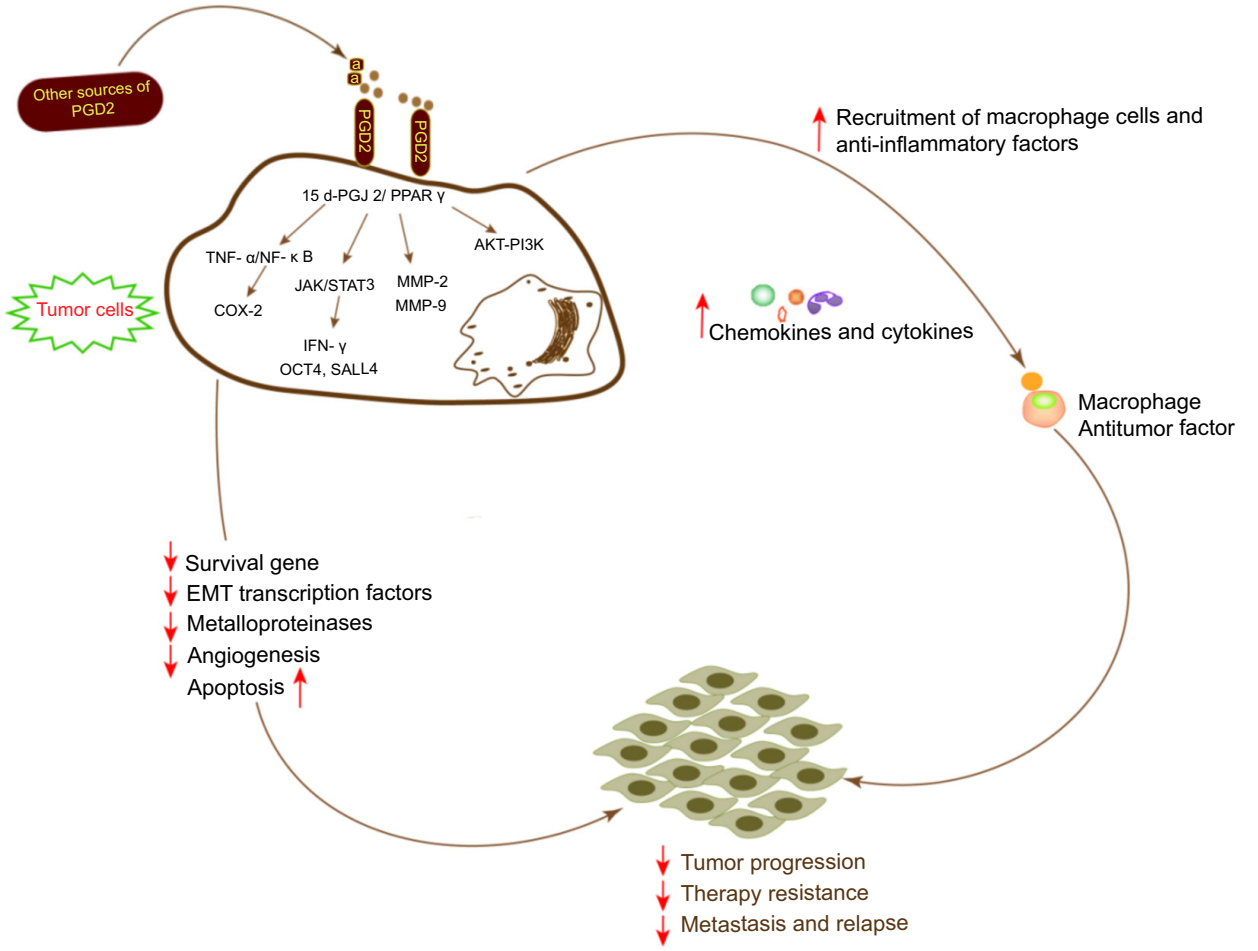
**Anticipated mechanism of action of PGD2 in antitumour.** TNFα: Tumor necrosis factor alpha; COX-2: Cyclooxygenase-2; NF-κB: Nuclear factor-κB; JAK 2: Janus kinase 2; STAT: Signaling, transcription activator factor; MMP-2: Matrix metalloproteinase-2; PI3K: Phosphoinositide 3-kinase.

In general, the PGD2/PTGDR2 pathway is considered to be a signaling cascade that inhibits cancer cell survival, proliferation, and migration. Furthermore, its tumor suppression pathways are complex and diverse, and involve direct mechanisms acting on tumor cells, as well as indirect mechanisms that remodel TME (Table 1 and Figure 3).

In vivo mouse models and in vitro cellular assays have shown that PGD2-associated signaling is critical for inhibiting cancer cell growth, proliferation, and migration in different tumor cell types. For example, PGD2 suppresses cell migration and invasion to inhibit in vitro and in vivo ovarian cancer cell growth in a dose-dependent manner, thereby increasing the mice’s survival [90]. Moreover, mast cell-derived PGD2 inhibits vascular leakage and regulates TNF-α production, thereby remodeling TME, and limiting tumor progression [29]. In gastric cancer, activation of PGD2/PTGDR2 signaling pathway inhibits the JAK/STAT3 signaling pathway and downregulates the expression of stemness-associated proteins, such as octamer-binding transcription factor 4 (OCT4), leucine-rich repeat-containing GPCR-5 (LGR5), Sal-like protein 4 (SALL4), and NANOG, which inhibits the self-renewal ability of gastric cancer cells [20]. Furthermore, the negative correlation of PTGDR2 with mRNA expression of OCT4, NANOG, LGR5, and SALL4 in cancerous tissues also demonstrated the relevance of these in vitro results to gastric cancer. Additionally, the ability to inhibit PGD2’s tumor-suppressing effect after PTGDR2 knockdown indicates that the presence of PGD2 ligands is essential for PGD2’s tumor-suppressing effects [74]. Zhang et al. [20, 21] showed that PGD2 or PTGDR2 could inhibit the nuclear STAT3 expression and promote its cytoplasmic expression in SGC-7901 and HGC-27 gastric cancer cell lines, thereby inhibiting the self-renewal ability and differentiation of the tumor, as well as inhibiting tumor growth and drug resistance in vivo. The PGD2/PTGDR2 pathway inhibits the protein expression of the metalloproteinases, MMP-2 and MMP-9, key mediators for tumor invasion and metastasis. This reduces the ability of SGC-7901 and HGC-27 to migrate and invade in vitro and in vivo [21, 91]. Furthermore, Yoon et al. [92] revealed that the PGD2/PTGDR2 signaling pathway inhibits epithelial–mesenchymal transition (EMT) and reduces lung inflammation in pulmonary fibrosis. EMT is closely associated with inflammation, drug resistance, tumor growth, migration, and invasion. In normal or low serum cultured A549 lung cancer cells, PGD2 induced PTGDR2 expression and also regulated EMT by mediating TGF-β1 expression, which in turn affected cell proliferation, migration, and invasion [93]. Thus, EMT modulation can promote the tumor-suppressive effect of the PGD2/PTGDR2 pathway.

A study showed that in OCUM-2 MD 3, MKN-74, MCG-803 gastric cancer cell lines, PGD2 and 15-d-PGJ-2 inhibited cancer cell growth, migration, and invasion by activating PPARγ signaling pathway and promoted apoptosis [93–95]. Similarly, Jang et al. showed that in breast cancer, 15-d-PGJ-2 decreased the invasive capacity of breast cancer cells (MCF-7) by regulating MMP-9 expression, which was inhibited by NF-κB/AP-1 activation. Interestingly, the NF-κB and AP-1 binding sites are in the promoter region of the MMP-9 gene [96]. Furthermore, the PGD2/PTGDS-related signaling pathway inhibited MCF-7 cell proliferation and migration by decreasing TWIST 2 levels, whereas TWIST 2 overexpression reversed the inhibitory effect of PGD2. Moreover, PGD2 also inhibited the expression of ALDH1A1, a breast cancer stem cell marker, which inhibited breast cancer angiogenesis and the self-renewal ability of breast cancer cells [22]. Consistently, 15-d-PGJ-2 reduced the invasive capacity of pancreatic cancer tumor cells by inhibiting the expression of MMP-9 and MMP-2, activating caspase-8 and caspase-9, as well as inducing apoptosis [97]. Other studies have shown that PGD2 and 15-d-PGJ-2 binding with the PPARγ receptor increases the expression of phosphatase and tension homolog deleted on chromosome ten (PTEN). PTEN is a tumor suppressor gene and regulates the proliferation and differentiation of tumor cells, thus achieving anti-tumor effects. Moreover, its overexpression can inhibit the PI3K signaling pathway and Akt phosphorylation, which reduces pancreatic cancer cell’s ability to proliferation and invade while promoting apoptosis and cell differentiation [37, 98]. In non-small cell lung cancer, 15-d-PGJ-2 induces apoptosis in A549 cells via a cysteine-dependent pathway and maintains cellular homeostasis [26]. Whereas, in human serum albumin (HSA) cultures, PGD2 mainly, D12-PGJ 2, significantly enhanced cell viability [26]. There is no association between 15-d-PGJ-2 and HSA. 15-d-PGJ-2 might be the main stimulator of apoptosis by PGD2. 

In lung cancer, PGD2 acts as an anti-tumor angiogenic factor and limits the further development of tumors by restricting the expression of some pro-angiogenic factors such as TNF-α and VEGF. Furthermore, a study has shown that PGD2 induced apoptosis in a prostate cancer model of the mouse by regulating nitric oxide production in the TME [37]. Consistently, in prostate cancer, 15-d-PGJ-2 inhibits the growth and induces partial differentiation of cancer cells, whereas reducing in vitro PGD2 levels by using specific antibodies or its knockdown from the culture medium eliminates tumor cell growth inhibitory effect and restores tumor resistance. Furthermore, Koeffler indicated that prostate cancer cell line (PC-3) cultured with PPARγ ligand had reduced growth and morphological alterations [99]. Other PPARγ ligands also inhibited the development of prostate xenograft tumors in immunocompromised mice and the in vitro proliferation of prostate cancer cell lines [100]. Moreover, 15-d-PGJ-2 inhibited colitis and colon cancer in mice by suppressing NF-κB signaling via PPARγ signaling activation [70]. Moreover, in a mouse colon cancer model, NF-κB or Wnt/b-linker signaling in epithelial cells induces aberrant activation of PGE2 and TNFα, thereby causing tumorigenesis [101, 102]. Whereas PGD2 stimulation significantly inhibited NF-κB and TNF-α signaling, resulting in an anti-tumor effect [23]. Sakai et al. [103] showed that PGD2 inhibited the growth of the colon cancer cell line HCC-Y1 and induced cell-cycle arrest. 

In some tumor patients, PGD2 or PTGDR2 expression levels correlated with the occurrence of distant metastases. For example, PGD2 levels have been indicated to be significantly higher in colon cancer patients with liver metastases than in those without liver metastases. Similarly, in malignant melanoma patients, PGD2 levels were significantly correlated with lung metastases [85, 104]. Moreover, high expression of PGD2 in malignant melanoma inhibits tumor vascular permeability, angiogenesis, and EMT, resulting in anti-tumor effects [105]. Yu et al. demonstrated that in ovarian cancer, PGD2 and 15-d-PGJ-2 activated PPARγ to inhibit NF-κB pathway-activated COX-2 expression, thereby suppressing cell migration, invasion, and drug resistance [106, 107]. According to Qian et al. [51], in AML, PTGDR2 (referred to as GPR44 in the article) inhibited the KRAS-mediated MAPK and PI3K/AKT/mTOR signaling pathways, which promoted apoptosis of leukemia-initiating stem cells, whereas, PTGDR2 knockdown enhanced colony stimulation. In some chronic myeloid leukemia (CML) and AML, especially with high PTGDR2 expression in FAB subtypes M2, M3, and M6, patients showed better survival, suggesting that high PTGDR2 expression might be a target for leukemia therapy [51]. In addition, PGD2 in leukemia cells promotes apoptosis by increasing intracellular peroxide levels, promoting reactive oxygen species, and activating the caspase-3 signaling cascade [108]. Similarly, Liu et al. [109] investigated that activation of the PPARγ receptor by PGD2 and 15-d-PGJ-2 inhibited the growth of leukemia cells HL-60 and K562 and suppressed the expression of MMP-2, MMP-9, and extracellular matrix proteins, as well as reduced their adhesion and invasiveness. Furthermore, in the mouse leukemia model, PGD2 and 15-d-PGJ-2 promote apoptosis in CML and increase the survival rate [110].

It has been indicated that the PGD 2/PTGDR2 signaling pathway can act directly on ILC-2 to produce IL-5 and IL-13, thereby promoting the proliferation of normal hematopoietic stem and progenitor cells (HSPCs). Whereas, disruption of the PGD2-activated ILC-2-Treg axis by specific inhibition of PTGDR2 or IL-5 impedes the proliferation of malignant HSPCs [111]. The aforementioned studies indicate that an MSC-derived PGD2 activates the ILC-2-Treg axis, which may be a valuable therapeutic target for cancer and inflammation-related diseases.

## Conclusion

The aforementioned data revealed that PGD2 and PTGDR2 are key factors that inhibit tumorigenesis and cancer development by suppressing the proliferation, migration, and invasive properties of cancer cells. The first epidemiological study showed that non-steroidal anti-inflammatory drugs significantly reduced tumorigenesis and angiogenesis by inhibiting COX-2, which was also mediated by the PGD2/DP1 receptor signaling pathway [15, 37]. Furthermore, endogenous TNF-α signaling has been an important TME factor in pro-tumor development. Moreover, in TME, mast cell-derived PGD2 has limited the pro-tumor response by reducing TNF-α synthesis. Additionally, PGD2-mediated metabolites could remodel the TME and inhibit the migration of inflammatory cells such as neutrophils and macrophages, thereby enhancing the anti-tumor immune response and facilitating tumor treatment. 

Our unpublished data suggests that PGD2 activates autophagy and inhibits the self-renewal capacity of GCSC. Moreover, COX-2 produces PGE2, and unlike PGD2, which is generally regarded as an oncogenic factor, it constitutes the main PG secreted by tumors. Recently, it has been indicated that PGE2 via EP4 receptor signaling could increase cancer stem cell populations in intestinal tumors by activating PI3K and MAPK signaling [112]. In gastric cancer, PGE2 maintains the expression of stem cell-associated proteins (OCT4, CD44, and SALL4) and promotes inflammatory microenvironment formation. Consistently, in the stromal adipocytes of breast cancer, PGE2 has upregulated aromatase production to stimulate tumor cell proliferation and promoted cell survival by inducing Bcl-2, the anti-apoptotic protein, via Ras-MAPK signaling [112, 113]. Moreover, PGE2 has also activated the PI3K-Akt-PPARγ cascade in ApcMin/+ mice to promote colon tumor cell survival [114]. 

Immunologically, PGE2 has inhibited M1, promoted M2 macrophage phenotype, enhanced the differentiation and infiltration of myeloid-derived suppressor cells, and increased the proliferative capacity of Treg cells. As a biological mediator, PGE2 affects the classical oncogenic signaling pathways in tumor cells and shifts TME toward immunosuppression and evasion, thereby promoting tumorigenesis [115]. 

PGD2 had the opposite effect to PGE2 in inflammation and tumors. PGD2 and PTGDR2 could regulate the TME and related anti-cancer signals to exert anti-tumor effects. Moreover, PTGDR2 has demonstrated special therapeutic effects in leukemia diseases, where it promoted apoptosis and reduced the disease severity by modulating relevant signals. Therefore, targeting PGD2 or PTGDR2 rather than other receptors is a better strategy for anticancer therapy. In addition, PGD2 could be considered an anti-tumor protective agent and a good prognosis marker. 

Although the role of PGD2 and related ligands requires further attention, remodeling of the tumor immune microenvironment by TNF-α and NF-κB regulating, particularly by reducing the inflammatory cell infiltration, is an intriguing mechanism for resistance to some new immunotherapies.
